# High-frequency oscillation in early adult respiratory distress syndrome

**DOI:** 10.1186/cc13919

**Published:** 2014-06-12

**Authors:** Kahoko Taki, David T Huang

**Affiliations:** 1 Department of Critical Care Medicine, University of Pittsburgh, 642A Scaife Hall, 3550 Terrace Street, Pittsburgh, PA 15261, USA; 2 Department of Emergency Medicine, University of Pittsburgh, Suite 10028 Forbes Tower, Pittsburgh, PA 15260, USA; 3 The Clinical Research, Investigation, and Systems Modeling of Acute Illness (CRISMA) Center, University of Pittsburgh, 3550 Terrace Street, 6th floor, Pittsburgh, PA 15261, USA

## Expanded abstract

### Citation

Ferguson ND, Cook DJ, Guyatt GH, Mehta S, Hand L, Austin P, Zhou Q, Matte A, Walter SD, Lamontagne F, Granton JT, Arabi YM, Arroliga AC, Stewart TE, Slutsky AS, Meade MO; OSCILLATE Trial Investigators and the Canadian Critical Care Trials Group: **High-frequency oscillation in early acute respiratory distress syndrome.***N Engl J Med* 2013, **368:**795**–**805.

### Background

Previous trials suggesting that high-frequency oscillatory ventilation (HFOV) reduced mortality among adults with the acute respiratory distress syndrome (ARDS) were limited by the use of outdated comparator ventilation strategies and small sample sizes.

### Methods

*Objective*: The aim of the study was to compare HFOV with a conventional ventilation strategy that used low tidal volumes and high levels of positive end-expiratory pressure in patients with new-onset, early (<72 hours) moderate to severe ARDS.

*Design*: A multicenter, randomized, controlled trial was conducted in 39 ICUs in five countries.

*Setting*: Participants were adults with new-onset, moderate to severe ARDS.

*Intervention*: Patients were randomly assigned to HFOV targeting lung recruitment or to a control ventilation strategy targeting lung recruitment with the use of low tidal volumes and high positive end-expiratory pressure.

*Measurements*: The primary outcome was the 60-day in-hospital mortality from any cause. Secondary outcomes included ICU mortality, 28-day mortality, new barotrauma, new tracheostomy, refractory hypoxemia, refractory acidosis, mechanical ventilation duration in survivors, ICU duration in survivors, and hospitalization in survivors.

### Results

On the recommendation of the data monitoring committee, we stopped the trial after 548 out of a planned 1,200 patients had undergone randomization. The two study groups were well matched at baseline. The HFOV group underwent HFOV for a median of 3 days (interquartile range, 2 to 8); in addition, 34 of 273 patients (12%) in the control group received HFOV for refractory hypoxemia. In-hospital mortality was 47% in the HFOV group, as compared with 35% in the control group (relative risk of death with HFOV, 1.33; 95% confidence interval, 1.09 to 1.64; *P* = 0.005). This finding was independent of baseline abnormalities in oxygenation or respiratory compliance. Patients in the HFOV group received higher doses of midazolam than did patients in the control group (199 mg/day (interquartile range, 100 to 382) vs. 141 mg/day (interquartile range, 68 to 240), *P* < 0.001), and more patients in the HFOV group than in the control group received neuromuscular blockers (83% vs. 68%, *P* < 0.001). In addition, more patients in the HFOV group received vasoactive drugs (91% vs. 84%, *P* = 0.01) and received them for a longer period than did patients in the control group (5 days vs. 3 days, *P* = 0.01).

### Conclusions

In adults with moderate-to-severe ARDS, early application of HFOV, as compared with a ventilation strategy of low tidal volume and high positive end-expiratory pressure, does not reduce, and may increase, in-hospital mortality.

Ventilator management is a key component of acute respiratory distress syndrome (ARDS) management. Low tidal volume (6 ml/kg) ventilation has been shown to reduce mortality [1], raising the question of whether high-frequency oscillatory ventilation (HFOV) with even lower tidal volumes will be even more beneficial. The theory behind HFOV is to couple a very small tidal volume (approximately 1 to 2 ml/kg) with a very high respiratory rate (3 to 15 breaths/second), thus providing a constant mean airway pressure, and limiting alveolar overdistension and repetitive collapse and reopening injury.

In the United States, HFOV has been sporadically used in adult ARDS patients after the US Food and Drug Administration cleared it for pediatric use in the 1990s. A 2010 meta-analysis of HFOV versus conventional ventilation (CV) for ARDS pooled six trials. The meta-analysis included four adult trials, a total of 291 adult patients. The study concluded that HFOV might reduce hospital mortality in patients with ARDS compared with CV and is unlikely to cause harm [2]. However, some of the trials did not use lung-protective ventilation in the CV group [2-4].

HFOV has been used in ARDS patients as a rescue therapy. More recently, HFOV has been used as primary or secondary therapy early in the disease course, with some evidence of success [5,6]. However, the efficacy of HFOV in adults with early ARDS has not been proven. The current OSCILLATE trial sought to address this clinical question.

The OSCILLATE trial compared HFOV with a CV strategy that used low tidal volumes and high levels of positive end-expiratory pressure in patients with new-onset, early (<72 hours since onset of respiratory failure), moderate to severe ARDS. The primary outcome was hospital mortality. Patients discharged from hospital were assumed to be alive at 60 days. Secondary outcomes were mortality at other time points, barotrauma, organ dysfunction, duration of ventilation, and duration of ICU and hospital stay. The study cohort was relatively young (mean age 55 years) and had high acuity (mean Acute Physiology and Chronic Health Evaluation II score of 29). Approximately one-half of the patients had sepsis and/or pneumonia. Close to two-thirds were on vasopressors or inotropes at baseline. In-hospital mortality was 47 % in the HFOV group, as compared with 35 % in the control group (*P* = 0.005).

The OSCILLATE trial was a well-conducted, large, multicenter trial performed in several countries. The HFOV protocol was standardized based on pilot testing and consensus guidelines. There was concern for a learning curve associated with the use of HFOV, and most patients were enrolled at centers that were experienced with HFOV. However, subgroup analyses did not detect an interaction between treatment effect and the number of patients enrolled per site. Why then did the OSCILLATE trial demonstrate harm with HFOV?

First, the HFOV group received more sedatives and vasoactive drugs (91% vs. 84%, *P* = 0.01). Vasoactive drugs and neuromuscular blockers were administered for longer in the HFOV group. One mechanism for hypotension in HFOV is that high intrathoracic pressures lead to decreased preload and right ventricular dysfunction, and consequently hemodynamic compromise. Sedatives and opioids (most commonly midazolam and fentanyl) that can cause vasodilatation were administered for the same duration in the two groups (median 10 days), but during the first week the median doses were higher in the HFOV group.

Second, central venous pressure was used for volume assessment. The study used central venous pressure greater than 12 cmH_2_O as the cutoff point for adequate intravascular volume prior to initiating the study protocol. Central venous pressure is an imperfect surrogate for volume status and preload. Central venous pressure less than 12 cmH_2_O does not necessarily indicate inadequate volume status. Echocardiogram, pulse pressure variation, or other monitoring technologies could be used for more accurate intravascular volume monitoring during HFOV.

Third, the OSCILLATE trial used recruitment maneuvers and relatively high mean airway pressure; initial mean airway pressures were 30 cmH_2_O and often increased up to 38 cmH_2_O. The OSCAR trial did not use mandatory recruitment maneuvers and set the initial mean airway pressure to 5 cmH_2_O above the peak plateau pressure (mean mean airway pressure 26.9 cmH_2_O on day 1 and 25.1 cmH_2_O on day 3) and showed no harm with HFOV compared with CV [7]. In contrast to results of the OSCILLATE trial, there was no difference in 30-day mortality and no significant difference in the duration of inotropic agents or pressor use between HFOV and CV in the OSCAR trial.

The OSCILLATE trial found that HFOV did not reduce, and may increase, mortality in early ARDS patients. Further study of the ideal HFOV setting and appropriate patient population and hemodynamic monitoring during HFOV are warranted.

## Recommendation

Based on the results of this large multicenter randomized trial, HFOV is not recommended over CV with low tidal volume in early ARDS.

## Abbreviations

ARDS: Acute respiratory distress syndrome; CV: Conventional ventilation; HFOV: High-frequency oscillatory ventilation.

## Competing interests

The authors declare that they have no competing interests.
